# The fabric of life: what if mosquito nets were durable and widely available but insecticide-free?

**DOI:** 10.1186/s12936-020-03321-6

**Published:** 2020-07-20

**Authors:** Fredros Okumu

**Affiliations:** 1 Environmental Health & Ecological Sciences, Ifakara Health Institute, Ifakara, Tanzania; 2 School of Public Health, University of the Witwatersrand, Johannesburg, Republic of South Africa; 3 Institute of Biodiversity, Animal Health & Comparative Medicine, University of Glasgow, Glasgow, UK; 4 School of Life Science and Bioengineering, Nelson Mandela African Institution of Science & Technology, Arusha, Tanzania

**Keywords:** Insecticide-treated nets, Insecticides, Malaria, Untreated nets, Long-lasting untreated nets, Insecticide resistance

## Abstract

**Background:**

Bed nets are the commonest malaria prevention tool and arguably the most cost-effective. Their efficacy is because they prevent mosquito bites (a function of physical durability and integrity), and kill mosquitoes (a function of chemical content and mosquito susceptibility). This essay follows the story of bed nets, insecticides and malaria control, and asks whether the nets must always have insecticides.

**Methods:**

Key attributes of untreated or pyrethroid-treated nets are examined alongside observations of their entomological and epidemiological impacts. Arguments for and against adding insecticides to nets are analysed in contexts of pyrethroid resistance, personal-versus-communal protection, outdoor-biting, need for local production and global health policies.

**Findings:**

Widespread resistance in African malaria vectors has greatly weakened the historical mass mosquitocidal effects of insecticide-treated nets (ITNs), which previously contributed communal benefits to users and non-users. Yet ITNs still achieve substantial epidemiological impact, suggesting that physical integrity, consistent use and population-level coverage are increasingly more important than mosquitocidal properties. Pyrethroid-treatment remains desirable where vectors are sufficiently susceptible, but is no longer universally necessary and should be re-examined alongside other attributes, e.g. durability, coverage, acceptability and access. New ITNs with multiple actives or synergists could provide temporary relief in some settings, but their performance, higher costs, and drawn-out innovation timelines do not justify singular emphasis on insecticides. Similarly, sub-lethal insecticides may remain marginally-impactful by reducing survival of older mosquitoes and disrupting parasite development inside the mosquitoes, but such effects vanish under strong resistance.

**Conclusions:**

The public health value of nets is increasingly driven by bite prevention, and decreasingly by lethality to mosquitoes. For context-appropriate solutions, it is necessary to acknowledge and evaluate the potential and cost-effectiveness of durable untreated nets across different settings. Though ~ 90% of malaria burden occurs in Africa, most World Health Organization-prequalified nets are manufactured outside Africa, since many local manufacturers lack capacity to produce the recommended insecticidal nets at competitive scale and pricing. By relaxing conditions for insecticides on nets, it is conceivable that non-insecticidal but durable, and possibly bio-degradable nets, could be readily manufactured locally. This essay aims not to discredit ITNs, but to illustrate how singular focus on insecticides can hinder innovation and sustainability.

## Background

Insecticide-treated nets (ITNs) have been a major component of malaria prevention campaigns for the past three decades. With a history nearly as old as modern civilization [1–3], bed nets are the most ubiquitous malaria prevention technique and one of the most effective [4]. When available and correctly used, their benefits are primarily derived from bite prevention and from killing or repelling mosquitoes.

**Fig. 1:**
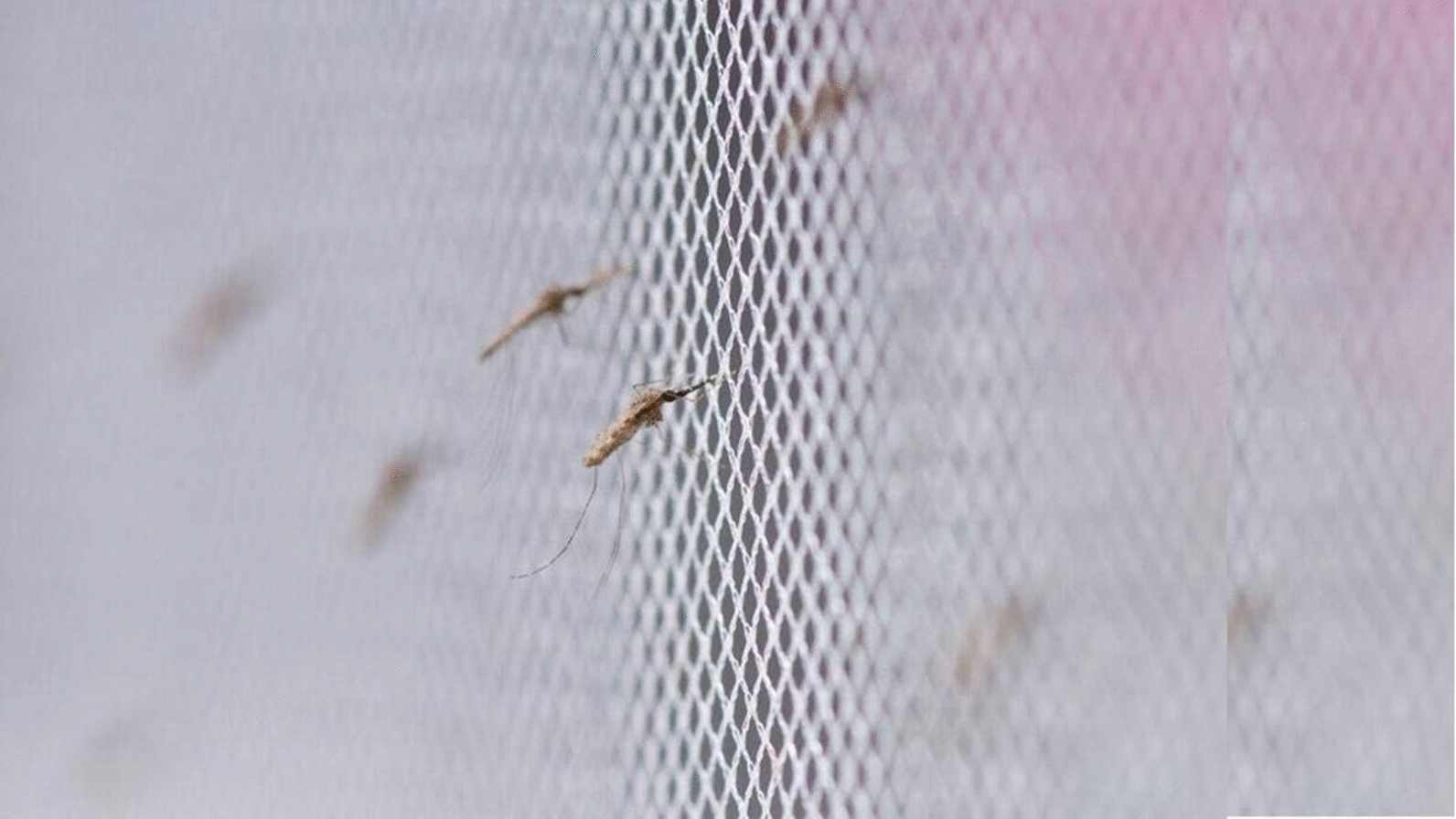
The Fabric of Life. Intact nets, if available and are consistently used, offer substantial benefits whether or not they kill mosquitoes. As mosquitoes become more strongly resistant to public health insecticides, the overall value of the nets comes increasingly from their ability to prevent biting rather than their ability to kill mosquitoes. This raises the question of whether bed nets, as long as they are durable and widely available, must also be insecticidal

The ability of nets to prevent biting primarily depends on how long the nets remain intact, which in turn is dependent on the physical durability and integrity of the nets. On the other hand, their ability to kill or repel mosquitoes depends on the chemical content of the fabric, and the degree of mosquito susceptibility to these insecticides. Where nets are sufficiently repellent, they may also directly prevent biting. Historical transitions in the bed net industry notwithstanding, one key question is whether the nets must always have insecticides on them, and whether non-insecticidal nets still have impact. The ecological and epidemiological transitions in malaria landscapes, notably the spread of insecticide resistance in malaria vectors, make this question even more relevant (Fig. 1).

The purpose of this article is to describe the modern history of mosquito nets for malaria control and carefully examine biological and epidemiological justifications for adding insecticides onto the nets. It reviews historical evidence and trends in the use of untreated and treated mosquito nets as well as key entomological and epidemiological outcomes. The article also examines biological observations of mosquito life cycle processes and how the mosquitoes respond to interventions inside dwellings. Lastly, it presents arguments for and against adding insecticides to nets, under multiple contexts, namely: (a) pyrethroid resistance, (b) personal versus communal protection, (c) outdoor-biting, (d) need for local production and (e) current global policies.

## A brief history of nets, their use and treatment with insecticides

In addition to being the top malaria prevention tool today, the mosquito net is also one of the oldest [3]. Its invention most likely preceded raised sleeping beds, and was primarily used for preventing bites of mosquitoes and other insects in periods not limited to sleeping hours. Lindsay and Gibson reviewed evidence from as early as 5th century BC [1], and found examples of nets used against insect bites and evil spirits, for privacy and as status symbols. It is possible that before the discovery that *Anopheles* mosquitoes transmitted malaria in late 19th century [5, 6], the use of nets was not linked to prevention of insect-borne diseases. Herodotus spoke of Egyptian fishermen sleeping under nets to avoid gnats [7], but there is no evidence they associated any insects with malaria.

**Fig. 2:**
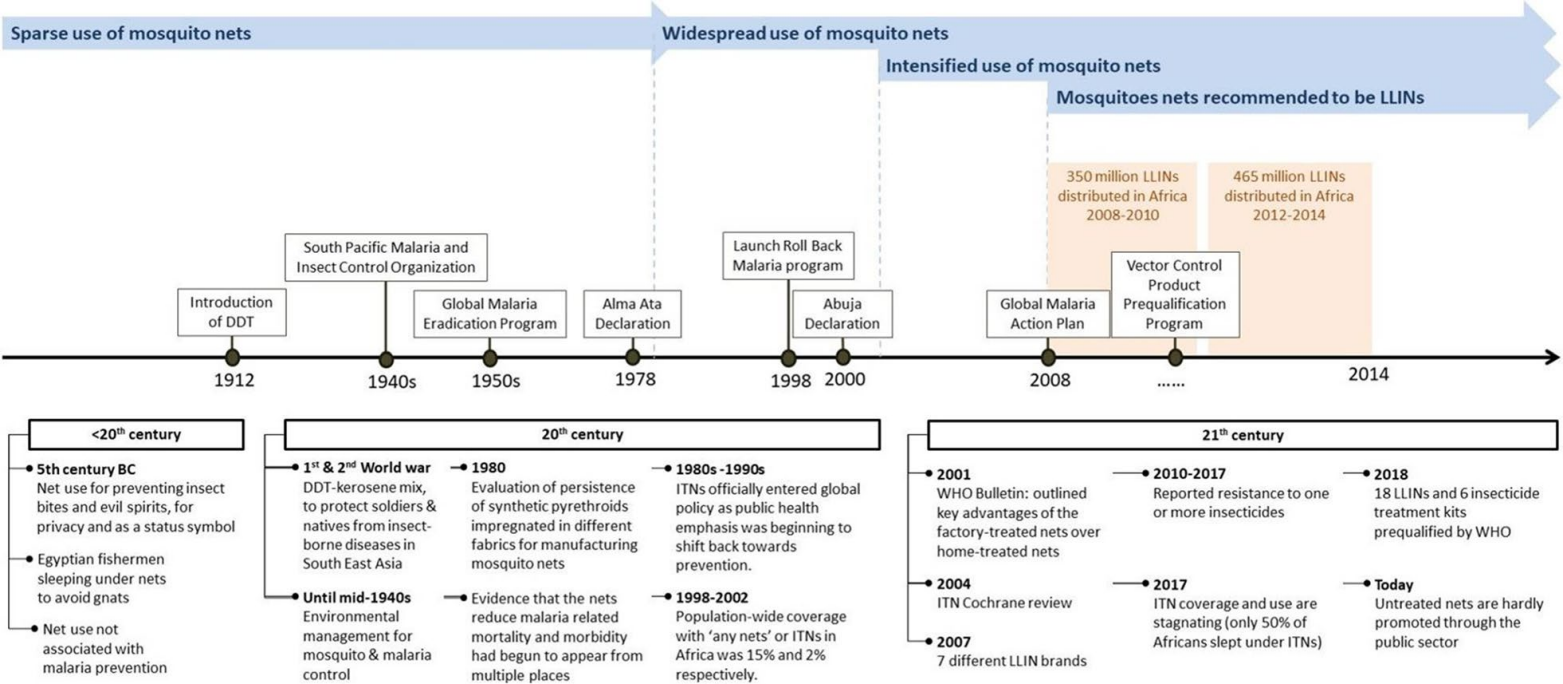
An evolutionary account of mosquito nets used in malaria control, showing key dates, developments and relevant health policies (Image created by Manuela Runge, Northwestern University, USA)

Until mid-1940s (Fig. 2), control of mosquitoes and malaria depended on environmental management, protective housing, proper sanitation, biological control, and use of toxic larvicides [8]. Nets, whether treated or untreated, and house spraying with insecticides were still rare, except in isolated cases [1, 9–11]. Malaria prevention changed dramatically following the second World War, when insecticide-based methods were first used on a large scale against adult mosquitoes. Introduction of DDT, which quickly became the main weapon, was the most significant development at the time [12, 13]. It is also around this period when bed nets and jungle hammocks were first treated with insecticides, to protect soldiers and natives from malaria and other insect-borne diseases in south east Asia [14]. Other reports indicate that insecticidal treatment of netting fabric was also practiced in the Soviet Union around this time [2, 3]. In the 1940s, the US military, fighting against Japanese forces in the Pacific, established the South Pacific Malaria and Insect Control Organization (SPMICO), to address epidemics of tropical diseases. In 1944, in a series of measures against adult mosquitoes, they began experimenting with bed nets and jungle hammocks impregnated with 5% DDT mixed in kerosene to control *Anopheles farauti* [14]. Unfortunately, for another four decades, the mosquito nets remained rare and mostly restricted to wealthy households. Moreover, the few available at the time were untreated.

In 1980, Hervy and Sales described an experimental evaluation of the persistence of synthetic pyrethroids impregnated in different fabrics for manufacturing mosquito nets [15]. A few years later, records from West Africa suggest that a team led by French scientist, Pierre Carnevale, may have pioneered the modern-day ITNs for mosquito control, as they were the first to systematically evaluate ITNs in controlled experiments inside huts. Darriet and Carnevale compiled a World Health Organization (WHO) report in 1984 on the efficacy of permethrin-impregnated intact and torn mosquito nets against malaria vectors [16]. According to this report, the ITN work had been motivated by previous applications of insecticide-treated fabrics and screens against tsetse flies, vectors of African trypanosomiasis [17, 18] and black flies, vectors of onchocerciasis [16]. The 1984 report concluded that permethrin-treated nets did not completely protect humans from anopheline mosquitoes, but substantially reduced man-vector contact to such a degree that the nets, even if damaged, could still significantly prevent malaria in at-risk populations [16].

Widespread use of mosquito nets for malaria prevention, only began in late 1980s and early 1990s, two decades after the first attempted global malaria eradication campaign, which had relied mainly on IRS with DDT [19]. As public health emphasis began shifting back to prevention, with a renewed role for vector control (in line with the 1978 Alma Ata declaration [20]), ITNs officially entered the global health policy on malaria control [3, 21]. Evidence that the nets reduce malaria-related mortality and morbidity had begun to appear from different countries, including The Gambia and Tanzania [22–24]. Roll Back Malaria (RBM), launched in 1998, became a major advocate for intensified use of ITNs. In 2000, African Heads of States met in Abuja, Nigeria, and endorsed a set of malaria targets, including 60% coverage of at-risk populations with ITNs and IRS [25].

As more trials were conducted, a Cochrane review concluded that ITNs reduced malaria cases by 39 to 62% and overall child mortality by 14 to 29% [26]. Shortly thereafter, the ITN targets were revised upwards to 80% under the RBM Strategic Plan 2005–2015 [27], before finally shifting to universal coverage (which originally meant coverage of 100% bed spaces), following adoption of the 2008 Global Malaria Action Plan (GMAP) [28]. In the GMAP, the WHO also expressly recommended that all new nets distributed be long-lasting insecticide-treated nets (LLINs), rather than conventionally-treated or untreated nets. LLINs were defined as ITNs that can withstand at least 20 washes and 3 years of use in field settings, a definition that remains unchanged [29]. Today, untreated nets, hand-treated nets or re-treatment kits are hardly promoted through the public sector.

## Trends in distribution and use of treated and untreated nets from the year 2000 onwards

Up to 624 million ITNs, most of them LLINs, were delivered to users globally between 2015 and 2017, up from 465 million delivered between 2012 and 2014 [30]. Of these, 552 million were distributed by national malaria control programmes (NMCPs), 83% in sub-Saharan Africa. The majority of these nets (85%) were provided to users for free in mass campaigns aimed at achieving high coverage and equity. Today, the number of ITNs distributed has exceeded two billion and is expected to continue growing [31]. To sustain high coverage, all these nets need to be replaced at least every 3 years.

Analysis of bed net use before 2010 (the approximate year when LLINs first became the more dominant form of ITNs, and also the year when ITNs achieved the greatest annual impact on malaria-related child deaths in Africa [32]), reveals two especially interesting trends. First, was the transition from non-insecticidal nets to ITNs, followed by increased acquisition of LLINs over ordinary ITNs starting 2006. Second was the gradual increase in coverage of ITNs (including LLINs) in malaria endemic countries and adoption of universal coverage targets.

## Transition from untreated nets to insecticide-treated nets

Until 1990s, mosquito nets were used mostly for physical protection against bites [1], and were mainly untreated, i.e. non-insecticidal [33, 34]. The untreated nets were never distributed in large scale campaigns, such as the mass campaigns done today. In fact, even epidemiological trials with such nets were small scale and were gradually abandoned when the impact of treated nets on malaria transmission was first found to be higher [24, 35–37]. The untreated nets provided modest protection when used properly and when in good condition [38–41], but their efficacy rapidly deteriorated. Often, they were rendered unprotective if torn, because infective mosquitoes could enter and bite the occupants [42]. These challenges were particularly serious in those early days since net designs, knitting patterns and fabric strength did not withstand frequent use or handling in the households, and were therefore easily torn.

Then came the conventional ITNs, which were bundled either at the factory or after dispatch with a sachet of pyrethroids. It had been demonstrated as early as 1992 that insecticide treatment could restore efficacy of torn nets [42]. Users were required to treat or retreat their nets with the insecticides approximately two times every year to sustain bio-efficacy. Home-based re-treatment intensified in the early- and mid-2000s but was operationally difficult to sustain, thus derailing ITN strategies [40]. Without regular re-treatment, the insecticidal efficacy of these nets rapidly declined through natural decay and repeated washings [43, 44].

To increase effectiveness, new manufacturing technologies were developed utilizing longer-lasting fibers, better knitting patterns and more enduring treatment techniques to produce long-lasting insecticide treated nets (LLINs), which were more durable and robust [45, 46]. In modern LLINs, the insecticide is either incorporated within the fibers or coated on surfaces of the fibers. According to the WHO, LLINs must retain bioefficacy, as measured by cone bioassays, without re-treatment for at least 20 washes and three years of use [47]. In a 2001 letter [48], Guillet et al., outlined key advantages of the factory-treated nets over home-treated nets as: (1) requiring no re-treatment, (2) consuming less insecticide and (3) having reduced environmental impacts from insecticides released in natural waters. The manufacturing industry responded by producing nets that were durable and had extended bioefficacy. This allowed an extraordinary level of scalability, which would otherwise have been impossible with the conventionally-treated ITNs and retreatment kits.

After the WHO Pesticide Evaluation Scheme (WHOPES) established the testing and evaluation guidelines for LLINs, Olyset^®^ [49], PermaNet^®^ [50] and Interceptor^®^ nets [51] became the first three LLINs to be recommended in 2001, 2004 and 2007, respectively. By 2008, distribution and sales of nets had significantly shifted from ordinary ITNs to LLINs in nearly all malaria endemic regions, though it was slower in India and some south-east Asian countries [52, 53]. By the time WHOPES transformed in 2017 to the current Vector Control Product Prequalification mechanism (WHO-Vector Control PQ), it had recommended 15 LLIN brands, all impregnated or coated with deltamethrin, alpha–cypermethrin or permethrin [54]. Eight of these had full recommendation, while seven had interim status. By February 2020, under the new PQ system, 20 LLINs had been prequalified [55].

## Increasing household and population coverage with treated and untreated nets

Utilization of ITNs in Africa increased steadily subsequent to the Abuja declaration by African heads of state in 2000 [25], but proportions of households or people using the nets (either treated or untreated) remained dismal until 2003 [33, 34, 56]. For children under 5 years old, untreated nets coverage may have reached 20% in a few countries (e.g. Guinea-Bissau, Mali, Sao Tome and Principe, The Gambia, Comoros, Tanzania, Chad and Benin), but coverage with ITNs remained generally below 5% [33]. Only the islands Sao Tome and Principe and The Gambia had ITN coverage greater than 10% among under-fives in 2003. Monasch et al., estimated, based on 1998–2002 health surveys, that population-wide coverage with ‘any nets’ and ITNs in Africa was 15% and 2% respectively [56]. Fortunately, most malaria endemic countries had adopted ITN policies by 2005 [34], and the global community was increasingly supporting the net campaigns.

By 2004 great progress was being made as health authorities revitalized efforts towards equity, and novel delivery methods such as social marketing and mass distribution became popular [57–62]. In Malawi, there was 8% coverage with any net in 2000 but this had quickly risen to 36% with ITNs by 2004 [63]. From 2003 to 2004, ITN use among under-fives increased from 4.6 to 23% in Senegal, 10.2 to 16% in Tanzania and 6.5 to 23% in Zambia [34]. Other notable champions were Togo and Niger, where household ITN possession rose from 8 to 63% and 6 to 61% respectively [33, 34], and Eritrea which reached 63% ITN coverage by end of 2004 [64].

More than 127 million ITNs were distributed freely or at subsidized costs to people living in malaria risk areas over 3 years starting in 2004, and about 96 million of these nets went to Africa [65]. An important cause for the rapid availability and uptake of ITNs was the 2005 decision by the WHO, announced at the 2005 multilateral initiative on malaria (MIM) conference in Yaoundé, that the nets should be made available for free to all women and children under 5 years old (W. Takken, pers. commun.).

By 2007, when the revised WHO coverage target of 80% was already in place [27, 66], countries with 60% household ITN ownership now included also Kenya, Niger, Sao Tome and Ethiopia [65]. A particularly exemplary performer was Zambia, where ITN ownership rose by 38% from 2006, reaching 62% in 2008 [67]. In 2008, the RBM partnership launched the Global Malaria Action Plan [28], which targeted universal and sustained coverage of all at-risk-populations with preventive and curative measures, to achieve malaria elimination country by country. It was also clear at this time that Africa in particular needed free ITN distribution to maximize gains for the poor [10]. For prevention, GMAP primarily advocated the use of LLINs (as opposed to conventional ITNs) and IRS, though it also peripherally encouraged other methods, if supported by local evidence. Production, distribution and use of insecticides and LLINs grew exponentially. Approximately 730 million LLINs would be distributed globally between 2008 and 2010, of which 350 million went to Africa. By 2010, an increasing number of countries had met the previous targets [25, 27, 28, 66], and were considering universal net distributions as outlined in GMAP.

Percentage of African households with access to an insecticidal net reached 56% in 2014, then 67% in 2015 when 82% of people with nets actually used them [68]. Considering whole populations (other than just people with nets), proportions sleeping under ITNs had risen to 46% in 2014 and 55% in 2015 [68]. For children under five years, this proportion had risen from < 2% in 2000 to 68% in 2015 [68]. Unfortunately, ITN coverage and use began stagnating, and in 2017, only 50% of Africans slept under ITNs, while population with access was 56% [30].

These estimates were probably excessive given they were derived from surveys simply asking people whether they had slept under an ITN the previous night, but without direct verification. Moreover, the surveys did not capture the protection gaps occurring when people are outdoors or indoors but not under bed nets, both of which contribute significantly to residual malaria exposures [69, 70], and are estimated to cause an extra 10.6 million malaria cases annually even if universal coverage is achieved with ITNs and IRS [71].

## Evidence for insecticide-treated nets in malaria control

Field evidence for effectiveness of bed nets has increased tremendously since 1990s and today include large-scale simulations claiming, perhaps against the McKeown thesis [72], that ITNs contributed the largest share of gains against malaria since 2000 [4, 32]. Starting late 1980s and early 1990s, a cohort of young researchers working in multiple countries pioneered large-scale clinical trials demonstrating that expanded use of ITNs substantially reduced malaria cases and all-cause mortality among African children [24, 62, 73–75]. Alonso et al., suggested that ITNs in the context of primary health care had significant health benefits exceeding any other interventions [24]. These early studies heralded a major wave in global health, eventually cementing ITNs as the primary malaria prevention tool.

In Tanzania and Ghana, village-wide benefits were reported in late 1990s, when large-scale distribution through social marketing campaigns achieved very high coverage even in rural areas with intense malaria transmission [62, 73, 74]. Elsewhere in the holoendemic western Kenya villages, Hawley et al. confirmed that ITNs had mass community-wide impacts, even extending to neighbouring non-intervention sites (see section on ‘mass community effects’ below) [75, 76]. Further evidence was provided in a series of research papers by Lengeler et al., starting mid-1990s [77–80], culminating in 2004, with perhaps the most influential review of ITNs ever [26]. The review comprehensively examined effects of ITNs in multiple settings and concluded that the nets reduced malaria cases by 39–62% and all-cause child mortality by 14–29% [26].

Eisele et al. used the Lives Saved Tool (LIST) [81] to quantify likely impact of malaria interventions on child survival between 2001 and 2010 across 43 sub-Sahara African countries [32]. They estimated that 842,800 malaria related child deaths had been prevented by scaling up the interventions, equivalent to 8.2% decrease in deaths expected over that period if there had been no scale-up. In their estimates, 99% of reduction was directly attributable to ITNs [32]. In a later assessment of multinational campaigns, a team of researchers examined field surveys from several malaria endemic countries and their epidemiological transitions between 2000 and 2015, to estimate intervention effects [4]. They concluded that ITNs on their own, had contributed 68% of the ~ 660,000 clinical malaria cases averted between 2000 and 2015 [4]. More evidence for effectiveness of ITNs exists in different formats. For example, a WHO-commissioned five-county trial originally aimed at evaluating impact of insecticide-resistance on effectiveness of ITNs showed that persons sleeping under nets were better protected than those without nets [82].

## Evidence for untreated nets in malaria control

Before 2005, most nets in Africa were untreated, and ITNs (which at that time were ordinary as opposed to long-lasting versions) were mostly confined to areas with ongoing trials. Unfortunately, similar to the de-prioritization of alternative anti-malarial tools following the arrival of DDT in 1940s, the rise of ITNs let to stagnation of any further development or trials of untreated nets after 1990s. Untreated nets may have been initially very attractive for malaria prevention, but the far superior efficacy of insecticide-treated versions resulted in their subsequent relegation [41]. Other than the study by Snow et al. in The Gambia in 1980s [83], there were no large-scale randomized controlled trials similar to those conducted for ITNs. As a result the quantity of entomological or epidemiological evidence for untreated nets is far less than for ITNs. Nonetheless, several observational studies make it possible to assess benefits from the simple physical protection by these untreated nets when they were still dominant.

Clarke et al. compared the risk of malaria parasitaemia among young children sleeping with or without nets in 48 villages in The Gambia, where the nets were already widely used in 1996 despite being mostly untreated [84]. Using cross-sectional surveys, they concluded that untreated nets in good condition were associated with significantly lower prevalence of *Plasmodium falciparum* infection (51% protection). Children from the poorest families benefited the most (62% protection), leading the authors to suggest that control programmes should prioritize lowest income groups [84]. The study also showed that biting risk was not diverted from users of nets to non-users, and that population coverage was inversely correlated with *P. falciparum* prevalence, suggesting communal benefits of untreated nets. Elsewhere in Kilifi, coastal Kenya, Mwangi et al. [85] demonstrated that intact untreated nets prevented approximately 60% of malaria infections and 35% of clinical disease, relative to no nets. On the contrary, torn untreated nets prevented approximately 25% of infections, but had no effect on clinical disease [85]. In these early studies, the alternative was often home-treatment and retreatment of nets every few months, so authors frequently concluded that untreated nets would be a useful alternative.

More compelling entomological investigations were done in Papua New Guinea, where Hii et al. investigated relationships between area-wide coverage of untreated nets and prevalence of *P. falciparum* malaria in community-based surveys in six villages over 33 months [86]. Here, untreated nets significantly reduced malaria vector survival and infections with *Plasmodium* sporozoites. A decline in *P. falciparum* prevalence in humans was also explained by bed net coverage [86].

In some trial sites, retrospective evidence suggests that high coverage with untreated nets already delivered significant gains ahead of the widespread use of ITNs. In one example, Russell et al., disaggregated data on impact of untreated and treated nets from surveys conducted in Kilombero Valley, south-eastern Tanzania in 1990s and 2000s [87]. This was a historically holoendemic setting where malaria prevalence reached 80% in late 1980s, and consistently exceeded 60% in 1990s [88, 89]. Compared to the high transmission intensities of up to 1400 infectious bites per person per year (ib/p/y) in certain households in early 1990s, bed nets regardless of treatment status had achieved 18-fold decrease in transmission, reaching 81 ib/p/y by 2009. When combined effects of insecticide treatment and high bed net coverage were considered, there was an additional 4.6-fold reduction in transmission beyond that already achieved with the untreated nets alone [87].

In the 2002 investigation by Guyatt and Snow on the cost of not treating nets [40], they provided examples, from The Gambia [90] and Tanzania [62], where the re-treatment rates of nets were not perfect, but all-cause mortality reductions remained significant. For example, in Tanzania, there had been a 19% protective efficacy of untreated nets compared to 27% for treated nets [62]. Guyatt and Snow, therefore, concluded that untreated nets were at least half as protective as treated nets, though in that period the net fabrics were still weak and prone to rapid wear and tear [40]. They also showed that untreated nets would be equally as cost effective as net re-treatment programmes. Separately, in a 2002 review [41], Takken also noted that untreated nets offered considerable protection, but that such benefits were unsustainable unless the nets were properly used, maintained in good condition, and sufficiently large to avoid sleepers making continuous physical contact with the net fabric. He proposed additional trials on untreated nets, and stated that large and intact untreated nets should be reconsidered to ensure sustainability. He further argued that such nets could be made more available and cheaper over longer periods [41]. An earlier review by Choi et al., also concluded that nets alone, without any treatment could deliver at least half of the protection as conferred by ITNs [91], findings eventually corroborated by Lengeler in 2004 [26].

When intact untreated nets were directly compared to various LLINs and IRS treatments in experimental huts in Tanzania, the intact untreated nets offered similar levels of personal protection (preventing > 99% of indoor blood-feeding), as three pyrethroid-treated net brands (Olyset^®^, PermaNet 2.0^®^ and IconLife^®^ nets) although these treated nets also caused modest mosquito mortality, not exceeding 20% [92]. When high-coverage in rural Africa was simulated in silico, the intact untreated nets provided similar personal protection to all other insecticidal nets, and at least half of the total additional community-level protection accruable by the three LLINs [93]. Separately, a model based analysis depicted that durability of LLINs is an essential component of their cost effectiveness [94], and that programme managers should be willing to pay more for nets with longer lifespan. Though no comparison is available for similarly durable but non-insecticidal nets, the evidence above suggests that long-lasting untreated nets could be significantly impactful.

## How do mosquito nets actually work, and what do the insecticides contribute?

**Fig. 3:**
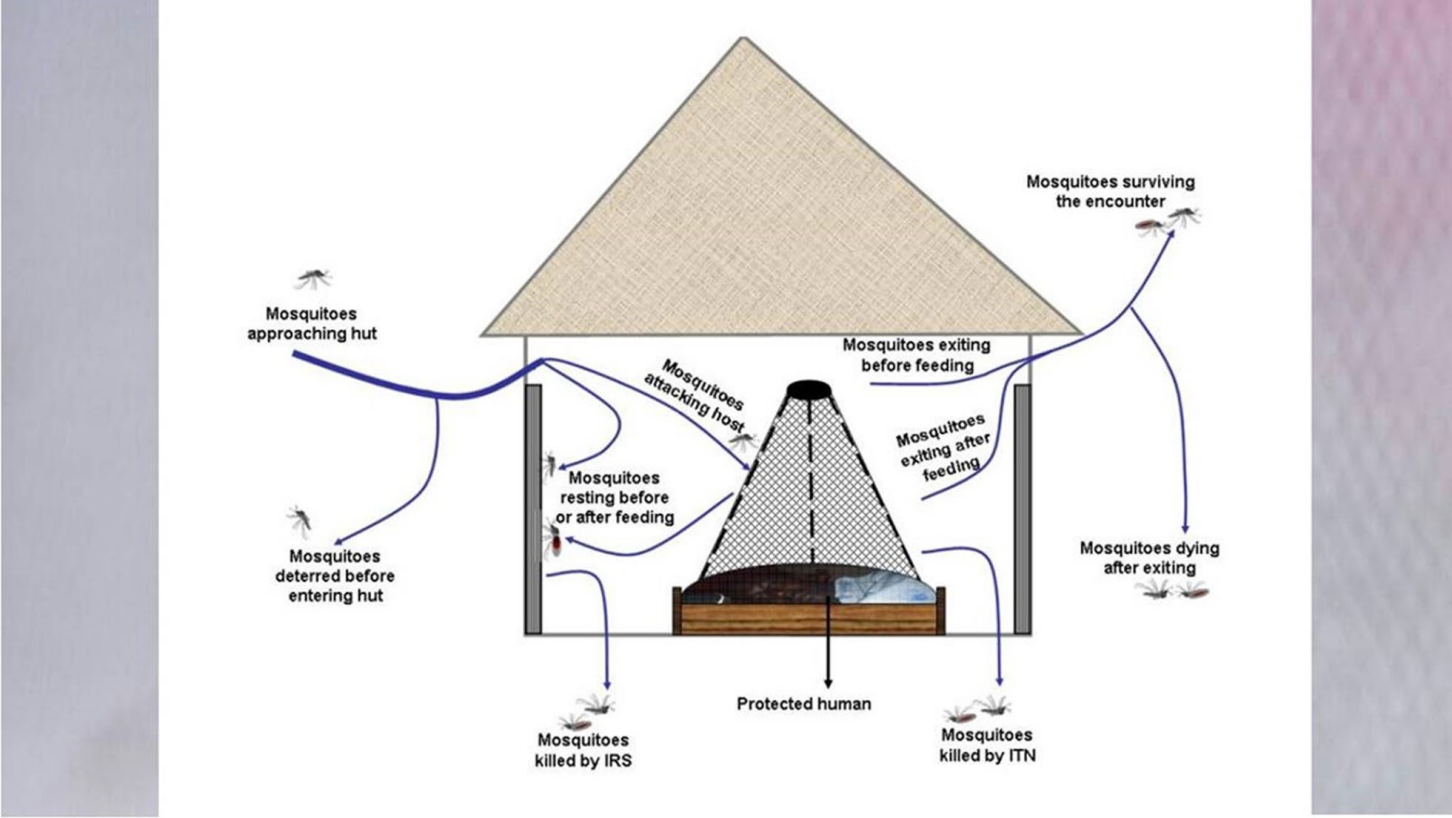
(Adapted from Okumu & Moore 2011 [100]: a diagrammatic representation of various effects of untreated or insecticide-treated nets (ITNs) and indoor residual spraying (IRS) on mosquitoes that enter or attempt to enter houses. Mosquitoes can be deterred and diverted before they enter houses, killed by the insecticides used on IRS or ITNs inside houses, or irritated so that they exit huts earlier than normal. This exit may occur before or after the mosquitoes have blood-fed, but both fed and unfed mosquitoes may die later after exiting, due to sub-lethal effects of insecticides. The net and IRS may also reduce mosquitoes’ ability to successfully take blood meals from the hut dwellers or to successfully transmit disease

The best way to assess how bed nets function is to consider important mosquito life cycle processes and their behavioural responses to interventions used inside human dwellings, e.g. ITNs and IRS (Fig. 3 and Tables 1, 2). During the host-seeking process, female *Anopheles* mosquitoes enter houses to obtain blood meals and/or rest on indoor surfaces. The full host-seeking process consists of two successive stages as follows: (1) non-host oriented kinesis, which involves arbitrary movements of the mosquito before it encounters any host cues, and (2) host-oriented taxis, involving directional movement once the mosquitoes detect the cues and begin moving towards the human host [95].

**Table 1:** Examples of trials showing properties of conventionally treated nets (ordinary home-treated ITNs) commonly used in Africa, on mosquitoes that enter or those that attempt to enter human huts

Insecticide	Country	Major vector species	Washing	Dosage (mg/m^2^)	Holes	Deterrence (%)	Bite prevention & feeding inhibition (%)	Toxicity (%)	Excess % exit	Reference
Alpha cypermethrin	The Gambia	*An. gambiae s.l*	Unwashed	100	Yes	0	92.0	94.0	–	[114]
			Washed	100	Yes	0	91.0	74.0	–	
	Tanzania	*An. arabiensis*	Unwashed	25	Yes	25.0	82.6	32.8	1.9	[108]^a^
		*An. gambiae & An funestus*	Unwashed	10	Yes	45.8	81.5	59.5	–	[115]
			Washed	10	Yes	27.9	67.7	24.8	–	
			Unwashed	20	Yes	21.2	68.5	63.4	–	
			Washed	20	Yes	13.6	66.7	43.5	–	
			Unwashed	40	Yes	11.4	79.1	50.1	–	
			Washed	40	Yes	44.2	79.2	43.5	–	
		*An. gambiae*	Unwashed	20	Yes	21.1	67.9	72.0	3.5	[106]
		*An funestus*	Unwashed	20	Yes	32.7	69.3	70.6	8.4	
		*An. gambiae*	Washed	20	Yes	0	29.9	69.6	6.0	
		*An funestus*	Washed	20	Yes	7.7	9.9	58.4	4.8	
Permethrin	Tanzania	*An. arabiensis*	Unwashed	200	Yes	33.7	72.0	49.8	–	[116]^b^
			Unwashed	200	No	20.6	61.0	41.9	–	
			Unwashed	80	No	10.6	71.2	–	28.3	[103]^c^
		*An. arabiensis*	Unwashed	25	Yes	35.3	85.8	15.2	5.9	[108]
			Unwashed	200	No	57.1	75.0	89.0	27.0	[103]
			Unwashed	1000	No	66.6	63.0	70.0	56.0	
		*An. gambiae & An funestus*	Unwashed	200	Yes	38.7	97.8	46.3	–	[107]
			Unwashed	200	Yes	20.5	82.2	29.8	–	
	Kenya	*An. gambiae*	Unwashed	500	No	15.0	83.9	–	50.8	[112]^e^
		*An. arabiensis*	Unwashed	500	No	0	66.7	–	13.9	
		*An. funestus*	Unwashed	500	No	35.7	85.9	–	49.6	
		*An. gambiae s.s*	Unwashed	500	No	94.6	–	–	–	[102]^e^
		*An. funestus.*	Unwashed	500	No	96.7	–	–	–	
	The Gambia	*An. gambiae s.l.*	Unwashed	5	Yes	33.0	96.3	74.0	2.0	[36]^d^
		*An. gambiae s.l.*	Unwashed	50	Yes	45.1	98.2	75.0	4.0	
		*An. gambiae s.l.*	Unwashed	500	Yes	69.9	98.7	79.0	10.0	
Lambda Cyhalothrin	The Gambia	*An. gambiae s.l.*	Unwashed	25	Yes	33.3	97.8	89.0	0	[36]^d^
	Tanzania	*An. gambiae & An funestus*	Unwashed	10	Yes	33.6	63.3	71.4	–	[115]
			Washed	10	Yes	31.8	54.8	61.3	–	
			Unwashed	20	Yes	32.6	63.3	74.8	–	
			Washed	20	Yes	23.0	62.3	56.0	–	
		*An. gambiae s.l.*	Unwashed	18	Yes	26.4	96.1	98.5	10.7	[117]
Deltamethrin	The Gambia	*An. gambiae s.l*	Unwashed	25	Yes	11	93	88	–	[114]
			Washed	25	Yes	–	87	74	–	
			Unwashed	500	Yes	60	98	72	–	
			Washed	500	Yes	–	87	54	–	
			Unwashed	25	Yes	22	98	86	–	
			Washed	25	Yes	0	87	87	–	
	Tanzania	*An. arabiensis*	Unwashed	25	Yes	30.7	81.4	33.0	2.5	[108]
		*An. gambiae*	Washed	25	Yes	22.5	89.0	69.0	6	[109]
			Unwashed	25	No	0	90.3	83.9	–	[105]
			Washed	25	No	0	91.2	70.2	–	
		*An. gambiae & An funestus*	Washed	25	No	0	95.2	88.0	–	

The effects are classified as deterrence, feeding inhibition, toxicity, and excess exit. This table includes a section of studies conducted in Africa, in areas where no resistance against DDT or pyrethroids had been reported. In studies where parameter values were not explicitly stated in the original publication, these values have been calculated from summary tables given in those original publications. *Deterrence* is calculated as the difference between number of mosquitoes entering treated huts and number entering control huts and is presented as a percentage of the number entering the control hut. *Bite prevention and feeding inhibition* is calculated as the percentage of all mosquitoes entering the treated huts that do not manage to feed. For purposes of uniformity, this formula was also applied to recalculate feeding inhibition for those studies where the authors had originally corrected the percentage feeding rates in treatment huts on the basis of feeding rates in control huts e.g. in Tungu et al. [109]. *Toxicity* on the other hand has been calculated as the percentage of mosquitoes entering the treated hut that die and *excess exit* is derived as the difference between percentage exit rates in sprayed and unsprayed huts, based on values presented in the original publications. The nets are grouped as per the active ingredients (insecticides) used to treat them. This table is adapted from Okumu and Moore 2010 [100] and the list of studies is inconclusive

^a^ In the study by Mosha et al. [108], the percentage mortality observed among mosquitoes collected in control huts was greater than 20%, therefore the toxicity values represented here are statistically corrected percentages

^b^ In studies by Lines et al. 1985 and Lines et al. 1987, the vector species are reported as *An. gambiae s.l.* though the original publications also had statements indicating that these mosquito populations were almost entirely *An. arabiensis* [103, 116]

^c^ Results represented in this raw from the study by Lines et al. [103] were obtained from tests of nets made of cotton rather than polyester as used in the rest of the studies

^d^ Deterrency and feeding rates in the Lindsay et al. 1991 paper were recalculated, by subjecting the log numbers presented in the original publication to a microsoft excel function (z = IMEXP) that returns the actual number of mosquitoes (z) as an exponential of a complex of numbers originally in x + y_i_ or x + y_j_ format

^e^ In the studies by Mathenge et al. [112] and Bogh et al. [102], the data used was based on pyrethrum spray catches done inside local huts and also from catches of exiting mosquitoes trapped using Colombian curtains [123] installed around village huts that were allocated (or not allocated) nets

**Table 2:** Examples of trials showing properties of different long-lasting insecticidal nets (LLINs) commonly used in Africa, on mosquitoes that enter or those that attempt to enter human occupied huts

Type^a^	Insecticide	Country	Major vector species	Washing	Holes	Deterrence (%)	Bite prevention and feeding inhibition (%)	Toxicity (%)	Excess % exit	Reference
PermaNet 2.0™	Deltamethrin	Tanzania	*An. gambiae*	Unwashed	Yes	20.6	90.0	95.0	0	[109]
				Washed	Yes	18.9	91.0	85.0	2	
			*An. gambiae*	Unwashed	No	0	93.0	97.7	–	[105]
				Washed	No	0	96.4	96.6	–	
			*An. gambiae & An funestus*	Unwashed	No	0	93.4	85.5	–	[105]
				Washed	No	0	98.2	93.0	–	
PermaNet 3.0™	Deltamethrin	Tanzania	*An. gambiae*	Unwashed	Yes	41.2	97.0	95.0	0	[109]
				Washed	Yes	22.8	90.0	94.0	0	
Interceptor™	Alpha cypermethrin	Benin	*An. gambiae s.l*	Unwashed	No	22.5	90.0	95.0	22.5	[51]^b^
				Washed	No	22.5	90.0	95.0	22.5	
		Tanzania	*An. gambiae*	Unwashed	No	0	88.0	93.0	15.0	
				Washed	No	0	82.0	73.0	15.0	
			*An. funestus*	Unwashed	No	0	–	76.0	–	
				Washed	No	0	86.0	60.0	–	
			*An. gambiae s.l.*	Unwashed	No	–	93.0	88.0	–	
				Washed	No	–	79.0	84.0	–	
			*An. funestus*	Unwashed	No	–	67.0	–	–	
				Washed	No	–	61.0	96.0	–	
Olyset™	Permethrin	Tanzania	*An. arabiensis*	Unwashed	Yes	0	96.3	11.8	25.6	[108]^c^
			*An. gambiae & An funestus*	Unwashed	No	5.4	87.2	56.0	–	[107]
				Unwashed	No	0	90.3	55.0	–	
				Washed	No	0	97.2	70.0	–	
				Unwashed	No	0	80.4	49.0	–	
			*An. gambiae*	Unwashed	Yes	0	40.9	62.7	7.2	[106]
			*An funestus*	Unwashed	Yes	28.9	49.9	73.9	1.4	
			*An. gambiae & An funestus*	Washed	No	0	81.1	57.5	–	[107]^d^
			*An. gambiae*	Washed	Yes	0	0	40.0	5.9	[106]^d^
			*An funestus*	Washed	Yes	30.8	0	58.9	4.2	

The effects are classified as deterrence, feeding inhibition, toxicity, and excess exit. This table includes a section of studies conducted in Africa, in areas where no resistance against DDT or pyrethroids had been reported. In studies where parameter values were not explicitly stated in the original publication, these values have been calculated from summary tables given in those original publications. *Deterrence* is calculated as the difference between number of mosquitoes entering treated huts and number entering control huts and is presented as a percentage of the number entering the control hut. *Bite prevention and feeding inhibition* is calculated as the percentage of all mosquitoes entering the treated huts that do not manage to feed. For purposes of uniformity, this formula was also applied to recalculate feeding inhibition for those studies where the authors had originally corrected the percentage feeding rates in treatment huts on the basis of feeding rates in control huts e.g. in Tungu et al., 2010 [109]. *Toxicity* on the other hand has been calculated as the percentage of mosquitoes entering the treated hut that die and *excess exit* is derived as the difference between percentage exit rates in sprayed and unsprayed huts, based on values presented in the original publications. This table is adopted from Okumu and Moore 2010 [100] and the list of studies is inconclusive

^a^ PermaNet 2.0™ is a 00% polyester LLIN coated with 55–62 mg of synthetic deltamethrin per square metre. PermaNet 3.0™ on the other hand is a mosaic-style LLIN specifically designed for the control of insecticide resistant mosquito populations. Its side panels, which unlike PermaNet 2.0™ have strengthened borders, are made of deltamethrin-coated-polyester (with approximately 118 mg/m^2^ of deltamethrin), while the top panel is made of monofilament polyethylene fabric into which a higher dose of deltamethrin (approx. 180 mg/m^2^) and approximately 1100 mg/m^2^ of a synergist, piperonyl butoxide (PBO) are incorporated. This synergist inhibits mixed function oxidases, which are known to be associated with pyrethroid resistance. PermaNet 3.0™ is also manufactured by Vestergaard Frandsen, Denmark. Interceptor™ is a long lasting insecticidal net made of polyester coated with alpha cypermethrin (200 mg/m^2^). It is manufactured by BASF, Germany. Finally, Olyset™ is made of a polyethylene netting (150 deniers), that is impregnated during manufacture with synthetic permethrin at a concentration of 2% (equivalent to 1000 mg of active ingredient per square metre). It is manufactured by A to Z company, Tanzania

^b^ The results for Interceptor™ nets evaluation in Benin are reported in the WHO report in very general terms as follows: high mortality (above 95%), high blood feeding inhibition (above 90%), 15–30% deterrence and 10–35% increase in exophilly [51]. Values reported in this table are therefore estimated as minimum mortality (95%) minimum feeding inhibition (90%), mean deterrence (22.5%) and mean excess exit (22.5%)

^c^ In the study by Mosha et al. 2008 [108], the percentage mortality observed among mosquitoes collected in control huts was greater than 20%, therefore the toxicity values represented here are statistically corrected percentages

^d^The data represented in these specific rows were collected from studies where the Olyset™ nets tested had already been in use for 4 years [107] or 7 years [106]

As the mosquitoes navigate the human odour plumes from within the house, their path is modulated by: (1) house design features e.g. whether the eave spaces, doors and windows are open or screened [96–98], (2) host biomass or household occupancy, which determine extents and strength of the odour plumes [99], and (3) responsiveness of mosquitoes to insecticidal treatments on nets or indoor surfaces, with maximum effects of ITNs observable against fully susceptible vectors. Mosquitoes not immediately killed eventually exit alive, with or without sub-lethal effects, to continue other life cycle processes (Fig. 3).

## Diversion (or repellence)

Mosquitoes attempting house entry may be diverted before entering. This is the first stage at which nets may be effective if they are treated with insecticides that deter or repel mosquitoes, as was common with hand-treated ITNs in the days before LLINs [36]. The best way to measure diversion is in standardized experimental huts where all factors including innate attractiveness of the huts can be kept constant. The numbers of mosquitoes entering houses with specific net types (treatment group) and those entering houses without a net (control group) are recorded, and the differences calculated as proportions of catches in the controls. However, to assess pre-entry diversion due to actual insecticides on nets, the control should consist of untreated nets. Reports that ITNs deter mosquitoes from houses had been mostly observed in hand-treated nets rather than factory-treated LLINs (as reviewed by Okumu and Moore [100]), and also in some experimental hut evaluations of the new generation nets containing synergists [101].

Differences in study designs however make it difficult to draw conclusions. In one example, deterrence associated with hand-treated pyrethroid-based ITNs against mosquito vectors was observed using permethrin at either 500 mg/m^2^ or 1000 mg/m^2^ on unwashed nets [36, 102, 103]. In studies using pyrethroid-susceptible *Anopheles* in Tanzania, a long-lasting net treatment product, ICON Maxx (Syngenta, Switzerland), consisting of a slow-release capsule suspension (CS) of lambda-cyhalothrin and binding agent, achieved higher deterrence than conventional hand treatment of nets using the same insecticide, suggesting possible effects of the binding agent [104]. For LLINs nonetheless, such deterrent effects have been minimal, hardly exceeding 20% [51, 92, 105–109]. Separately, direct observations of *Anopheles* mosquitoes in a wind tunnel showed no excitorepellent effects when the mosquitoes were exposed to deltamethrin-treated nets [110]. However, one study in Burkina Faso showed significant pre-entry deterrence by LLINs having permethrin and deltamethrin, with up to ten-fold lower mosquito densities in LLIN huts compared to control huts [111].

Mosquitoes can also be diverted post-entry. This includes induced exits where the vectors are irritated by insecticides on the ITNs. The excess exit is measured by comparing proportions of mosquitoes caught at different times of night exiting huts with ITNs and huts with non-insecticidal nets. ITNs that irritate mosquitoes will cause earlier-than-normal exit. Data from past experimental hut and field studies show that this excess exit was often absent or minimal except with unwashed permethrin-treated nets [36, 103, 112]. However, where dominant vectors are opportunistic, e.g. *Anopheles arabiensis* which readily blood-feed on non-humans and can bite outdoors, most net types including untreated nets can cause early exit [92]. Mosquitoes that are unsuccessful in blood-feeding attempts leave early to find alternative blood sources elsewhere.

## Bite prevention and inhibition of blood feeding

This is possibly the most important function of bed nets and the main reason that even non-insecticidal nets can be effective. It is primarily determined by physical integrity, durability and actual use of the nets, and is assessed by measuring proportions of mosquitoes found inside houses (indoors or upon exit) that are not freshly blood-fed. Therefore, where nets are intact, large enough to cover the bed and are properly used, bite prevention can be near 100%, guaranteeing complete personal protection during sleeping hours.

In addition to the bite prevention, blood feeding inhibition can also occur due to physiological responses of mosquitoes to the insecticides on nets [113]. Despite differences in trial designs, significant bite prevention, exceeding 80% has been observed with untreated nets, nets treated with varying pyrethroid doses, washed and unwashed nets, holed and intact nets, and against multiple *Anopheles* species in multiple sites across Africa [103, 105, 106, 108, 109, 112, 114–117]. Even where vectors were resistant to pyrethroids and their mortality highly compromised, actual bite-prevention remains high, and very few mosquitoes successfully blood-feed as long as the nets are in use [118–120].

## Toxicity to mosquitoes

Mosquitoes that make physical contact with the ITN itself or with chemical vapours from the net could be killed if they acquire sufficiently lethal doses of the active ingredients. These mortality effects are purely of insecticide origin, and are not observable with untreated nets. By removing potentially infectious vectors from circulation, highly mosquitocidal nets can protect both the actual users and non-users.

ITN toxicity is measured by counting mosquitoes that die within the hut or after exiting the hut within a reasonable time period (usually 24 h), and expressing this as a proportion of collected mosquitoes that entered the hut. This is compared to similar counts and proportions from control huts with untreated nets. For best results, mosquitoes exiting the huts should not be held captive nearby for extended periods, as this may over-estimate percentage mortality due to overexposure to insecticide vapours. This was a common problem in early experimental hut designs, where catches were held until morning either in exit traps or special verandas attached to the treated huts [121–123]. Fortunately, recent advances in hut design and study protocols can minimize these challenges [124]. For example, in Ifakara Experimental Hut designs, first described in 2012, mosquitoes were retrieved by exit interception traps every few hours to avoid excessive exposure [124].

One important question is whether the ecological and epidemiological conditions that enabled massive ITN-associated gains, notably the widespread susceptibility of *Anopheles* to pyrethroids, still persist. Generally, ITN-associated mortalities are highest where vectors are physiologically susceptible and bite indoors during sleeping hours. Historically, in the pre-resistance age, indoor mortality commonly exceeded 70% across east and west Africa [103, 105, 106, 108, 109, 112, 114–117]. These effects were short-lived for hand-treated ITNs, due to rapid break down and decay of the insecticides, but arrival of LLIN technologies significantly extended the bio-efficacies. However, multiple studies have since demonstrated that where malaria vectors are either physiologically resistant to pyrethroids or readily bite outdoors, the insecticide-induced mortality can be strongly attenuated or diminished [92, 117, 119, 120, 125–127]. In Tanzania, mortality of moderately-resistant *An. arabiensis* exposed to permethrin and deltamethrin LLINs did not exceed 19.5% in experimental huts, although blood-feeding remained below 1% for 6 months [92]. The mortality rates were expected to continue declining as local *Anopheles* became more intensely resistant.

## Delayed effects

Delayed effects are commonly observed on mosquitoes that previously entered houses and successfully blood-fed or were diverted post-entry, but which did not die within 24 h. Where nets are insecticidal, mosquito survival can be significantly compromised if they exit after receiving sub-lethal doses. The effects are also observable where resistant mosquitoes are exposed to doses that would typically kill susceptible populations, an observation sometime associated with continued benefits of ITNs under pyrethroid-resistance situations [128]. Besides pyrethroids, other products used on nets such as chlorfenapyr [129, 130] and pyriproxyfen [131, 132] are functionally slow-acting and cause delayed mortality. Other than chemical toxicity, the reduced mosquito survival may also result from delayed blood-feeding and longer host-seeking cycles, occasioned by high coverage of treated or untreated nets in concert with environmental factors such as predation.

## Mass (community-level) effects

While bed nets primarily offer personal protection to users, they also protect non-users as well as other users in the same communities. These effects are accruable via multiple mechanisms, namely: (a) mass-killing of malaria mosquitoes, (b) reduced availability of humans to infected-*Anopheles*, b) reduced availability of infected humans to *Anopheles* and (c) reduced survival of mosquitoes during foraging.

If the vector populations are sufficiently susceptible, ITNs can cause major declines in vector populations and malaria transmission [133]. In addition to susceptibility, community-level effects of nets are also dependent on net coverage and behaviours of dominant vector species. In a cross-sectional survey in rural Tanzania in the early 2000s, Abdulla et al. examined prevalence of anaemia in communities with sub-universal ITN coverage during social marketing campaigns [73]. One year into the campaign, when 52% of children were already using ITNs, children living in areas of moderately high coverage were half as likely to have moderate or severe anaemia and had lower splenomegaly irrespective of their ITN use [73].

Separately, in the mid 2000s when the WHO coverage target was still 80% ITN coverage prioritizing children and pregnant women, Killeen et al. argued that broader population coverage was being overlooked at the expense of the demographic targeting [134]. To justify a more rational ITN distribution beyond just the vulnerable groups, they developed in silico simulations and tested these using data from multiple sites to estimate coverage thresholds, at which individual protection would become equivalent to community-level protection [134]. They showed that even 80% coverage of children and pregnant women gave only limited protection and equity for vulnerable groups, but just 35–65% coverage of all adults and children could achieve equitable community-wide gains equivalent to or greater than personal protection [134].

Perhaps the best evidence of the community-wide ITNs benefits was from the study done in early 2000s in Asembo-bay, western Kenya, a high transmission area then dominated by *Anopheles gambiae* sensu stricto (*s.s.*) and *Anopheles funestus* [75]. The study showed substantial protective effects of ITNs, including reduced child mortality, reduced anaemia, and lower parasite prevalence, even in homes lacking ITNs but within 300 m of homes with ITNs [75]. The non-user gains were correlated with proportions of nearby households having ITNs.

Community-level effects of nets are also accruable from combined effects of high population coverage and delayed host-finding. Irrespective of insecticide content, high coverage with nets will reduce availability of human hosts to mosquitoes [135], and increase duration of host-seeking, thus compromising their survival and reproduction. Where species are highly anthropophagic, e.g. *An. gambiae s.s.* and *An. funestus* [136, 137], high coverage with even untreated nets reduce blood-feeding, vector survival and parasite transmission. In Papua New Guinea, infection prevalence in humans was influenced more by bed net coverage among neighbours than by personal protection [86]. Infections in *Anopheles* were also inversely driven by percentage coverage. Moreover, the study showed that vectors may choose alternative blood hosts if humans used nets (zoophagy slightly increased in areas with high coverage of bed nets), though most dominant vectors still preferred humans [86]. It may therefore be necessary to have a complementary metric of bed net evaluation that relies on bite prevention as the key attribute.

Differential effects of nets on species may also cause proportionate shifts in vector populations. ITNs and IRS effectively control *An. gambiae s.s.* and *An. funestus*, which predominantly bite humans and rest indoors [126, 138, 139], but low-to-moderate transmission can persist and remain poorly-responsive to further increases of intervention coverage [92, 93, 126, 140, 141]. In some settings, residual transmission is increasingly mediated by vectors that readily bite people outdoors [138, 142–145]. This could also limit the overall impact of indoor interventions against malaria [94], except in settings where the dominant vectors, despite biting mostly outdoors, still go indoors at least once during their lifecycle [146, 147].

## Bed nets and insecticide resistance: why use pyrethroid-treated nets in places where mosquitoes are resistant?

Resistance to common public health pesticides is widespread in major malaria vectors across Africa and other regions. Of the 80 WHO member countries that provided resistance data for the period between 2010 and 2017, 68 reported resistance to at least one insecticide in major malaria vectors, and 57 had resistance to two or more insecticides [30]. Yet malaria vector control still relies primarily on LLINs (mostly treated with pyrethroids) and IRS.

It is therefore critical to assess justification of pyrethroid nets in areas with widespread resistance and whether the resistance profiles can compromise effectiveness of the ITNs. In 2010, Ranson et al. [148] noted that very few studies had assessed epidemiological impact of resistance pyrethroid, and the available evidence come with multiple confounders, making the findings difficult to interpret. Unfortunately to date, this situation has improved only slightly due to: (1) lack of comparator sites without any resistance, (2) the multiplicity of resistance mechanisms in mosquitoes, (3) variations in intensities of resistance across settings and vector populations, and (4) reluctance to roll-out other ‘potentially-inferior’ products (e.g. durable untreated nets) in place of LLINs, as controls in field trials.

Since the report by Ranson et al. [148], a large multi-country WHO-coordinated study has been completed in Sudan, Kenya, India, Cameroon and Benin to quantify potential loss of effectiveness of ITNs (and IRS in Sudan) due to decreased susceptibility of malaria vectors to insecticides [82, 149]. The study included 279 clusters across the five countries and enrolled 40,000 children with 1.4 million follow-up visits. The investigators assessed malaria prevalence and incidence in areas with low or high resistance as measured by standard WHO assays [150]. Their expectation was that if resistance was counter-effective, there would be higher incidence and higher prevalence of malaria in areas with low mosquito mortality (i.e. high resistance clusters), compared to areas with high mortality (low-resistance clusters). However, upon final analysis, ITN users had lower prevalence and incidence than non-users across all areas, but there was no association between resistance and either prevalence or incidence.

The authors concluded that irrespective of resistance, people in malaria endemic areas should continue using ITNs to reduce their risk of infection. Though not incorrect, this conclusion was unresponsive to the key research question, which was whether resistance was associated with loss of ITN effectiveness and increased malaria burden. A more accurate conclusion, given the findings, would be that resistance was not associated with loss of effectiveness of ITNs against malaria. The authors also ignored an alternative interpretation equally justified by their data. That is, the bed nets remained effective across all settings regardless of whether or not they sufficiently killed mosquitoes. This selective interpretation of findings was probably justified by a desire to avoid disrupting the supply chains of existing vector control products.

One missed opportunity in this study was the lack of a study arm with untreated nets, which would have further clarified the relevance of the chemical content of ITNs. There were also other concerns, in particular the apparent lack of power to detect differences between sites. This was crucial given the high variations in densities of *Anopheles*. The study also did not assess intensities of the resistance, and instead relied on percentage mortality values in standard WHO susceptibility assays to rank areas with high *versus* low level resistance. Lastly, it did not assess community protection, and instead relied on household-level protection when assessing the interventions. Nonetheless, there were substantial reductions in malaria prevalence in Sudan, when additionally using non-pyrethroid IRS relative to nets-only locations. This indicates that the lost-killing effect from pyrethroid nets in scenarios with resistance could be pronounced, and also that it may be more prudent to limit the use of insecticides to just IRS and instead keep nets as non-insecticidal.

In Benin, Asidi et al. [151] investigated whether nets still offered protection in two areas, one in the north where *An. gambiae* were susceptible and another in the south where *An. gambiae* were strongly resistant to pyrethroids. They determined that ITNs provided similar levels of personal protection as untreated nets in communities where resistance was high, regardless of physical conditions of the nets [151], suggesting that intact durable nets at high coverage could provide substantial cover, potentially mitigating against the threat of resistance. In Malawi, Lindblade et al. followed a cohort of children (6–59 months) in households with at least two ITNs, in areas having pyrethroid resistant *An. funestus* associated with elevated oxidases [152]. They found that malaria incidence among ITN users was 1.7 infections/person/year (ib/p/y) compared to 2.1 ib/p/y among non-ITN users, and that ITN use reduced incidence by 30% compared to no nets despite the resistance [152].

Overall, the available field evidence appears to suggest that insecticide resistance strongly diminishes effectiveness of IRS, but that its affects on ITNs are either unclear or minimal. In a famous malaria control programme on the borders of South Africa and Mozambique, a switch from DDT (to which prevailing *An. funestus* were susceptible) to deltamethrin (against which the vectors rapidly became resistant) resulted in higher malaria burden, but this was reverted when DDT was re-introduced [153]. Elsewhere in Malawi, Wondji et al., observed that selection of pyrethroid-resistance over 3 years did not increase malaria in children in areas with ITNs alone or ITNs plus pyrethroid IRS, but also that IRS did not yield expected gains under similar resistance profiles [154].

In Burundi, where the vectors had high frequency of *kdr*-genes usually associated with pyrethroid resistance, malaria episodes, vectors densities and transmission intensities were highly impacted when pyrethroid-ITNs and IRS were combined [155, 156]. A plausible explanation for these differences was that the benefits of ITNs, especially at high coverage and as long as they remain intact, are constituted primarily by bite prevention, which persists despite loss of susceptibility to insecticides. Ranson et al. [148] explained this phenomenon using data from a trial in northern Côte d’Ivoire, where *kdr*-allelle frequency in predominant *An. gambiae* was above 80%. In that trial, pyrethroid-based ITNs reduced both malaria transmission and clinical incidence in children by more than half, compared to controls without nets [157]. Ranson et al. argued this was the first clear-cut example, where ITNs continued to work despite high resistance in major vectors, and that the absence of physical barrier effects in control group might have led to an overestimation of ITN impact against *kdr*-mosquitoes [148].

It is, therefore, essential that nets should remain intact and be consistently used at high coverage, regardless of their ability to kill mosquitoes. In a more direct appraisal, Paaijmans and Huijben recently suggested removal of insecticides from bed nets and instead reserving these for other interventions, such as IRS [158].

## Importance of sub-lethal effects, coverage and physical integrity of bed nets used in settings with pyrethroid resistance

Though pyrethroid resistance is now common-place across Africa and susceptible *Anopheles* populations increasingly rare [159, 160], bed nets are still credited with most of the gains accrued against malaria [4, 32]. Other than the barrier effects, another explanation for why ITNs remain effective despite resistance is that the sub-lethal effects of insecticides are sufficient to maintain the impact. Since current LLINs have longer-lasting fabrics than traditional untreated nets, the combination of physical integrity and sublethal impacts could possibly outweigh any negative impacts of pyrethroid resistance. It is, therefore, essential to examine the potential of these sub-lethal effects alongside effects of population-level coverage and physical integrity of the nets in areas where direct toxicity of the nets to mosquitoes is severely weakened.

Thanks to advances in quantitative ecology, Bayesian state-space models were recently fitted with laboratory data and used to demonstrate how delayed mortality of female *Anopheles* exposed to ITNs, could cut the malaria transmission potential [128]. Though such observations are yet to be verified in the field, the authors rightly concluded that the delayed mortality on strongly resistant mosquitoes does not diminish the threat of resistance, but instead gives additional account of why ITNs remain effective despite resistance. Separately, a malaria transmission model, also fitted with laboratory and experimental hut data, was used to illustrate how even low-level resistance could increase disease incidence due to reductions in mosquito mortality and communal protection [161]. These findings were also contrary to the WHO-backed field observations showing that ITNs remained effective despite phenotypically-observable resistance [82]. Churcher et al. proceeded to recommend switching to new ITNs that have both pyrethroids and chemical synergists, as a way to regain impact [162].

Overall, the findings of these two mathematical evaluations, by Viana et al. [128] and Churcher et al. [161] remain open to different interpretations on whether pyrethroid resistance has negative or null impacts on the epidemiology of malaria. It appears though that the communal protection associated with mosquitocidal effects of nets is lost at moderate pyrethroid resistance, and that under strong resistance, even the ability of insecticides to restore efficacy of torn nets is lost.

An important attribute of insecticide resistance is that it does not manifest as “all or none” phenotypes, but rather on gradients with certain thresholds beyond which impact is completely lost. Though these thresholds are difficult to determine in field settings, sub-lethal insecticides have been shown to remain marginally impactful either by reducing survival of older mosquitoes [163–166] or disrupting development of malaria parasites inside the mosquitoes [166–168]. Moreover, repeated contacts may also lead to higher mortality typically undetectable using standard resistance tests [169]. These effects probably also contribute to the observed continued effectiveness of ITNs in pyrethroid resistance settings. However, the extent of such sub-lethal effects and whether they are observable across all forms of insecticide resistance is unclear. When Alout et al. challenged different strains of *An. gambiae s.s.* with wild *P. falciparum* isolates from Burkina Faso, they observed that resistance had varied effects on vector competence, including possible increase in sporozoite prevalence [170]. Besides, in the full life cycle of ITNs, reductions in overall mosquito mortality will limit ITN bioefficacies and the communal benefits [161]. Once high intensities of resistance are reached, even the sub-lethal effects may be lost.

An alternative explanation for continued effectiveness of ITNs despite resistance—that at high coverage, efficacy is less dependent on insecticidal content than on physical integrity—has also been examined. In Mozambique, Glunt et al. observed in WHO bioassays that ITNs no-longer effectively killed resistant mosquitoes [171], but subsequent in silico simulations considering both coverage and sub-lethal effects concluded the nets would remain effective unless coverage dropped significantly [172]. In rural Tanzania, pyrethroid-treated nets did not kill moderately resistant *An. arabiensis* in experimental huts, but conferred high level personal protection through simple bite prevention [92]. In the Tanzanian trials, high proportions of the mosquitoes survived cone bioassays on the nets, and the bioefficacy further decayed rapidly within months [173]. Follow-up mathematical simulations showed that at population-wide coverage, there was limited additional impact of insecticidal over non-insecticidal nets on malaria transmission [93].

Strode et al. also reviewed impacts of pyrethroid resistance on efficacy of ITNs against African malaria vectors [174]. Their conclusion was rather equivocal, but none of the many studies they examined categorically demonstrated failure of ITNs in the face of resistance. Hemingway et al. [175] explained in reference to the Strode et al. article [174] that “although some forms of pyrethroid resistance were clearly affecting entomological indicators such as blood-feeding and survival of mosquitoes, the quality of data, variability of experimental designs, and inconsistencies in methods of resistance measurement had made it impossible to assess effects on malaria transmission” [175]. What is uncontestable from all the studies and reviews is that while ITNs may lose their toxicity to mosquitoes under resistance scenarios, they remain highly impactful if they are intact and are properly used at scale. This implies that pyrethroid treatment may not be a universal necessity, but rather supplementary.

The multiplicity of effects accruable from bed nets, including physical and chemical barrier-effects, is clearly a challenge for scientists wishing to assess impact of resistance on malaria transmission [176]. Moreover, since insecticide resistance is never a binary “all or none” phenomenon, LLINs may retain some effective mosquitocidal efficacy, especially in older mosquitoes, further complicating the desire to isolate the physical barrier from the sub-lethal effects so as to assess them separately. More importantly however, developers should recognize that instead of singularly overemphasizing the need for new insecticides as suggested by some experts [11, 175], an alternative response to the resistance problem may simply be to ensure that bed nets are accessible, durable and properly used, even if non-insecticidal. New insecticides can then be developed for other forms of vector control, such as IRS but not bed nets. It has been demonstrated that resistant mosquitoes can survive up to 1000-times the concentration of insecticides that kill susceptible populations [127]. Such strongly-resistant mosquitoes may naturally incur major survival and fitness costs in nature [177], but are unlikely to be killed directly by insecticidal nets. In some settings across Africa, this gap is compounded by increased proportions of malaria exposure occurring either outdoors or indoors before bed time [70, 71].

In the early days before bed nets were of the long-lasting versions, it was known that insecticides would ensure the nets remained effective even after being torn [42]. More recent studies in western Kenya have also shown that bio-efficacies of ITNs with impaired physical integrity (measured based on the number of mosquitoes collected inside bed nets and the proportion of mosquitoes killed in cone bioassays) declined in areas of pyrethroid resistance [178]. Today, the physical integrity of nets has improved considerably compared to early ITNs, so findings of mosquitoes inside nets should be interpreted as failures of both integrity and bio-efficacy, rather than just failures of bio-efficacy.

## Importance of durability and functional survival of nets in the context of resistance

Fundamentally, the strength of nets is typically determined by their bursting strength, which refers to the capacity of fabrics to maintain continuity when subjected to pressure by stretching in different directions. It is a function of fiber quality (measured as deniers), knitting patterns and types of polymers used [179]. One review on this subject concluded that bursting strength of knitted fabrics depend not only on fabric structures and fiber types, but also the yarns used [180].

Beyond physical strength, another important factor is the environmental conditions under which the nets are used, as well as actual use patterns in households. The overall durability of bed nets therefore varies between communities, households or usage. Improving the overall durability of bed nets therefore requires among other factors, the selection of fabric designs with adequate physical strength, using a tear-resistant knitting patterns and ensuring appropriate use practices and handling in households. Evidence-based considerations for net durability under actual use patterns in different field settings should therefore be a key factor for evaluating bed nets during both the prequalification and post-market stages. This is particularly important given the relevance of bite-prevention in areas where mosquito susceptibility to pyrethroids has decayed, and the inability of health authorities to regularly replace nets every few years.

Field evidence increasingly shows that current ITNs do not last as long as expected under real use settings, and that there is a wide variation in both bio-efficacy and durability in different settings [181–184]. In one study in Tanzania, scientists examined the durability of ITNs distributed by the government between 2009 and 2011 in eight districts [185]. They found that just 2 years after the distribution of the ITNs, only 39% of the nets were still present in the households and in serviceable condition. The rest had attritioned out of the houses, mostly because they were considered too torn to use. A separate analysis conducted 2 years after the 2011 mass distribution confirmed that households generally lost their nets faster than they acquired new ones [186]. In this second study, less than 25% of households had one LLIN for every two people, and population coverage was only 32%.

It is important to continue prioritizing net durability without compromising user acceptability and affordability. Unfortunately, the ITN market dynamics have lowered the unit prices for nets so much so that manufacturers may be unable to continue improving the net quality while also remaining competitive. The need to improve durability is therefore often confounded by among other factors, the need to retain competitive unit costs, and the excessive emphasis on bio-efficacy as the main indicator of bed net performance.

Some studies have started combining these indicators to estimate the overall functional survival of ITNs, and have shown that more durable nets are indeed more effective. In one example, Lorenz et al. [187] investigated the functional survival of different ITNs distributed in Tanzania. Using a randomized double-blind approach, they observed three different net types distributed to more than 3400 households and assessed whether the nets were still present and in serviceable condition after each subsequent year post distribution, and whether the nets provided adequate protection to volunteers sleeping under them. This study showed that the median functional survival was less than 3 years in all net brands tested (2.0 years for Olyset, 2.5 years for PermaNet and 2.6 years for NetProtect), and that this outcome was affected by accumulation of holes which often resulted in users discarding their nets. Lorenz et al. also estimated that in totality, the longer-lived nets were 20% cheaper than the shorter-lived nets, further emphasizing the importance of ITN durability when assessing cost-effectiveness [187].

## Could ITNs with multiple active ingredients restore insecticidal efficacy?

In response to the loss of insecticidal bio-efficacy due to pyrethroid-resistance, second-generation nets with additional active ingredients are increasingly under consideration. Examples of the new actives include the pyrrole, chlorfenapyr [188] and the insect growth regulator, pyriproxyfen [189], but there are also ITNs which incorporate the synergist, piperonyl-butoxide (PBO) [109, 162, 190]. Each additional chemical ingredient performs differently; chlorfenapyr disrupts the oxidative phosphorylation of mitochondria and requires the recipient mosquito to move to kill the vector. This makes assessments using current bioassays ambiguous [188, 191]. On the other hand, the synergist PBO inhibits the natural defences of mosquitoes against pyrethroids thus reinstating mortality effects [192]. Lastly, pyriproxyfen exerts multiple effects including disruption of aquatic development, reduced emergence of adult mosquitoes, sterilization of females, and reduced egg-laying by the females [193–195].

Large-scale trials of PBO- and pyriproxyfen-based nets have demonstrated higher benefits than regular LLINs in areas with pyrethroid resistance in Tanzania [162] and Burkina Faso [131], respectively, though the improvements in the latter trial were only modest. Given its design, which also included an “IRS plus LLIN” arm, the Tanzania study also surprisingly showed that in settings such as these, insecticide resistance may negatively impact LLIN effectiveness, but that these could be restored by using PBO nets [162]. More recently, another large-scale trial has been completed in 48 Ugandan districts with high pyrethroid resistance comparing PBO nets (i.e. Olyset Plus and PermaNet 3.0) with non-PBO nets (Olyset and PermaNet 2.0) [196]. The investigators measured malaria parasite prevalence among 2–10 year-olds, and demonstrated that any benefits accruable from the PBO nets over standard ITNs were marginal at 6 months (11% in PBO group *versus* 15% in non-PBO group), and null after 12 and 18 months. Surprisingly, the authors concluded, contrary to their own findings that the PBO nets were more effective, and even went further to suggest that their evidence should be adopted by the WHO to provide the “final” recommendation for PBO-Nets [196].

Attempts by manufactures to label these new net products as “resistance-bursting” remain unappreciated due to lack of direct entomological data against comparator LLINs under resistance settings, and the fact that such nets come in multiple formats with different modes of action [197]. Moreover, while ITNs with both PBO and pyrethroids can kill susceptible and pyrethroid-resistant vectors, evolutionary theory dictates that such advantages cannot be sustained indefinitely in any area under selection pressures. The synergists can make the insecticide treatments more toxic but they do not necessarily reverse the resistance.

There have also been questions on whether the efficacy improvements in new LLINs are caused by higher quantities of pyrethroids on the nets or by the additional chemical compounds. For instance, PermaNet^®^ 3.0 nets have not only PBO, but also higher doses of deltamethrin on the fibres than the predecessor version, PermaNet^®^ 2.0 [109]. The WHO has recently emphasized that pyrethroid-PBO nets should not be considered a tool for insecticide resistance management in malaria vectors. It is expected that developing and evaluating dual LLINs treated with non-pyrethroid insecticides may be a better option, though this would also be temporary under resistance pressures.

Other concerns have been on how long the synergists such as PBO used on nets, such as PermaNet^®^ 3.0 and Olyset^®^ Plus, will last under natural conditions [197, 198], the higher costs of the new nets, and overall inconsistencies of observed benefits of these nets over regular ITNs across settings [197]. Besides, some of the new actives used on the new nets, such as pyriproxyfen, are detoxified by the same enzyme systems as those that detoxify pyrethroids [199] and could potentially exacerbate resistance in malaria mosquitoes even at very low doses (Opiyo et al., unpublished). Lastly, despite the hype, the actual pace of innovation around these new ITN types remains slow, and there are no guarantees for evolution-proof formulations that could be deployed at cost in the short or medium term to stem insecticide resistance.

In summary, ITNs with multiple active ingredients or synergists may provide temporary relief in high transmission settings with pyrethroid resistance, but their field performance, costs, field longevity and the drawn-out innovation timelines do not justify continuing a singular focus on insecticides. Moreover, introducing new classes or combinations of insecticides will certainly lead to new resistance due to evolutionary selection pressures. On the other hand, it is unlikely that behavioural resistance (arising because mosquitoes cannot reach humans for blood feeding), would reduce protection of intact nets. Hence, the need re-examine the role of durable untreated nets is both desirable and urgent.

## Could long-lasting untreated nets be useful for resistance management?

The global plan for insecticide resistance management in malaria vectors (GPIRM) outlines a series of steps necessary to preserve effectiveness of current insecticide-based interventions [200]. This plan has a technical basis laid by global experts under WHO coordination [201], and recognizes that managing resistance will be complex due to the multiplicity of its underlying mechanisms and cross-resistance. Despite some initial progress made by countries, uptake of GPIRM has been poor due to limited availability of alternative control tools with new modes of action to complement existing ones [202]. Instead, most countries still rely principally on ITNs (containing pyrethroids) and IRS. Other important concerns include limited financing and deficiencies in human and infrastructural resources [202].

There are no viable approaches to stop resistance, so GPIRM chiefly relies on delay-tactics such as combinations, rotations, mosaics or mixtures of different insecticide classes [201], but pays limited attention to insecticide-free options such as untreated nets or house screening, or refuge strategies such as those used in agriculture [197, 203]. Moreover, unlike most other WHO-backed policies which rely heavily on large-scale field trials, GPIRM borrows heavily from experiences in agricultural crop protection, without any epidemiological evidence that the recommended resistance-management approaches will actually improve heath outcomes in human populations. One resistance management program that compared rotations, mosaics and single-use of insecticides of IRS against malaria vectors in Mexico did not yield any conclusive findings [204].

To alleviate the insecticide resistance pressure on vector populations, non-insecticidal nets could possibly play a role either in the rotations, e.g. rotating long-lasting untreated nets with LLINs, or in combinations, e.g. using the untreated nets in houses sprayed with effective IRS compounds. When Paaijmans and Huijben recently suggested the removal of insecticides from bed nets [158], their main justifications were that this strategy would allow faster response to pyrethroid-resistance, slow down the insecticide treadmill and permit more effective use of the available insecticides for other vector control interventions. Except in selected settings such as northern Tanzania [205], the current practice of combining LLINs and IRS offers limited additional or synergistic value relative to just LLINs [206]. Theoretically, replacing the LLINs with untreated versions could potentially maintain similar protection while minimizing resistance pressure, though such an approach is yet to be field-tested.

If such durable but untreated nets were available at lower unit costs, the savings could be used to increase coverage with high-quality non-pyrethroid IRS. Given that this has not been done, it is important to compare epidemiological outcomes when using untreated nets against similar quality fabric treated nets in different resistant settings. This should be followed by a cost effectiveness analyses to help countries determine which net product (untreated nets, pyrethroid-treated nets, PBO-based nets, or dual-active nets) is best suited for different settings. The WHO may thereafter consider prequalifying some brands of durable untreated nets for use as complementary interventions, e.g. alongside IRS with non-pyrethroids.

Another alternative, for of communities with reduced malaria risk, could be to deploy the long-lasting untreated nets as the main intervention in small designated zones near zones with insecticidal interventions, so as to encourage refuge mosquito populations that remain susceptible to insecticides. Such spatial mosaics using refuge strategies are already widely used in agriculture [197], where farmers cultivating “*Bt*-crops” [crops with *Bacillus thuringiensis* (Bt) derived transgenes that code for insecticidal proteins conferring pest resistance] are encouraged to plant small sections of their farms with non-transgenic crops to minimize risk of *Bt*-resistant pests [191].

The more progressive components of GPIRM however are the recommendations for longer-term programmes for supplementary tools in the medium term, and sustainable disease control practices in the long-term [200]. More durable untreated nets could potentially constitute a sustainable option for resistance management if carefully deployed in rotation, combinations or spatial mosaics with existing methods.

## Where are the WHO-approved mosquito nets currently manufactured, and what role can malaria endemic countries play?

By February 2020, the WHO had prequalified 20 LLINs and 6 insecticide treatment kits for use by endemic countries [55]. The list currently contains no untreated net, not even a long-lasting version of untreated nets (Table 3). There are 13 manufacturers producing the 20 approved LLINs, but only three have presence in Africa. An important question, therefore, is whether endemic countries have adequate and uninterrupted access to affordable nets at all times. Another is why the private sector markets for bed nets have stagnated in Africa despite the constantly growing demand, with two billion LLINs now delivered [31].

**Table 3:** Long lasting insecticide treated nets and insecticide treatment kits with WHO prequalification and recommendation for use by malaria endemic countries and procurement by national and international agencies. Source: WHO Vector Control Product Prequalification Program, as at February 2020 [55]

	Product	Company/Address	Manufacturing facilities		Active ingredients
Long-Lasting Insecticide Nets (LLINs)					
1	Olyset Net	Sumitomo Chemical Co., Ltd, Japan	Kinh 2A, Phuoc Lap, Tan Phuoc, Tien Giang, Vietnam	A to Z Textile Mills Limited Tanzania; Net Health Limited, Tanzania	Permethrin
2	Olyset Plus	Sumitomo Chemical Co., Ltd, Japan	Kinh 2A, Phuoc Lap, Tan Phuoc, Tien Giang, Vietnam	A to Z Textile Mills Limited Tanzania; Net Health Limited, Tanzania	Permethrin; Piperonyl Butoxide
3	Interceptor	BASF SE, Germany	Shanghai Gongtai Textile Co Ltd; China Taicang City, Jiangsu Province No.2, Fada Road	SunshineThailand Nonthaburi 11000, Office: 18/2 Moo 7 Rattanatibet Rd.,Bangkrasaw, Muang	Alpha-cypermethrin
4	Interceptor G2	BASF SE, Germany	Shanghai Gongtai Textile Co Ltd; China Taicang City, Jiangsu Province No.2, Fada Road	SunshineThailand Nonthaburi 11000, Office: 18/2 Moo 7 Rattanatibet Rd.,Bangkrasaw, Muang	Alpha-cypermethrin; chlorfenapyr
5	Royal Sentry	Disease Control Technologies, LLC, USA	Dean Superior Textile Co., Ltd., China	–	Alpha-cypermethrin
6	Royal Sentry 2.0	Disease Control Technologies, LLC, USA	Dean Superior Textile Co., Ltd., China	–	Alpha-cypermethrin
7	Royal Guard	Disease Control Technologies, LLC, USA	Dean Superior Textile Co., Ltd., China	–	Alpha-cypermethrin and pyriproxyfen
8	PermaNet 2.0	Vestergaard S.A, Switzerland	Vestergaard S.A. Place Saint Francois 1, CH-1003, Lausanne, Switzerland	10/10 Textile Joint Stock Company Production site n.1: 9/253 Minh Khai street, Hai Ba Trung district, 114034Hanoi, Vietnam	Deltamethrin
9	PermaNet 3.0	Vestergaard S.A., Switzerland	Vestergaard S.A. Place Saint Francois 1, CH-1003, Lausanne, Switzerland	10/10 Textile Joint Stock Company Production site n.1: 9/253 Minh Khai street, Hai Ba Trung district,114034 Hanoi, Vietnam	Deltamethrin and Piperonyl Butoxide
10	Duranet LLIN	Shobikaa Impex Private Limited, India	Shobikaa Inpex Private Limited SF No.558,559, Athur SIDCO Industrial Estate, Vennaimalai PO Karur-639 006, Tamilnadu, India	Shobikaa Inpex Private Limited SF No.37A/1, b&C,D,E Coimbatore Road, Thannerpandhal, Karur-2	Alpha-cypermethrin
11	MiraNet	A to Z Textile Mills Ltd, Tanzania	A to Z Textile Mills Ltd; Plot No.698, Net world Area, Dodoma road, Arusha, Tanzania	–	Alpha-cypermethrin
12	MAGNet	V.K.A. Polymers Pvt Ltd, India	V.K.A. Polymers Pvt Ltd (UNIT-1) 169/1,170/1,192/3 Balarajapuram Village,Veerarakkiam, Karur District, Tamil Nadu, India 639114	V.K.A. Polymers Pvt Ltd (UNIT-2 (EOU)) 1/79 Maduari By-pass Road (NH7), Sadiaya Goundan Pudhur, Kakavadi (PO), Kakavadi Village, Karur District 639003, Tamil Nadu, India	Alpha-cypermethrin
13	Veeralin LLIN	V.K.A. Polymers Pvt Ltd, India	V.K.A. Polymers Pvt Ltd (UNIT-1) 169/1, 170/1, 192/3 Balarajapuram Village,Veerarakkiam, Karur District, Tamil Nadu, India 639114	V.K.A. Polymers Pvt Ltd (UNIT-2 (EOU)) 1/79 Maduari By-pass Road (NH7), Sadiaya Goundan Pudhur, Kakavadi (PO), Kakavadi Village, Karur District 639003, Tamil Nadu, India	Alpha-cypermethrin & Piperonyl butoxide
14	Yahe LN	Fujian Yamei Industry & Trade Co Ltd, China	Heranba Industries Ltd 101/102, Kanchanganga, Factory Lane, Borivli (W), Mumbai-400 092 India	Agros Chemicals India Ltd. Jhaver Centre, Rajah Ananmalai Building, IV Floor,19, Marshalls Road, Egmore, Chennai- 600 008, India	Deltamethrin
15	DawaPlus 2.0 LLIN	Tana Netting FZ LLC, Dubai	Sheikh Noor-ud-Din & Sons 4 km, Kanha Kacha Road, off Ferozepur Road, Lahore, Pakistan	Rosie’s garment factory nig. Ltd; 49a Milverton Avenue, P.O. Box 920, Aba Abia state, Nigeria	Deltamethrin
16	DawaPlus 3.0	Tana Netting FZ LLC, Dubai	Sheikh Noor-ud-Din & Sons 4 km, Kanha Kacha Road, off Ferozepur Road, Lahore, Pakistan	Rosies garment factory nig. Ltd 49a Milverton Avenue, P.O. Box 920, Aba Abia state, Nigeria	Deltamethrin; Pyperonil butoxide
17	SafeNet	Mainpol GmbH, Germany	Jin Xun Ye (Huizhou) Textile Company Ltd (Main manufacturing facility) No.431 Bo Yuan Road, He Shan Village, Yuan Zhou Town, Bolou County, Huizhou City, Guangdong Province, China	Fujian Changle Xingcheng Synthetic Co. Ltd Baihu Section, Lianggang Road, Zhanggang Town, Changle City, Fujian Province, China	Alpha-cypermethrin
18	YorkoolLN	Tianjin Yorkool International Trading Co., Ltd, China	Tianjin Yorkool International Trading Co., Ltd.North of Yangliuqing Power Station, 104 National Road, Tianjin, China	Gaotang Xingyuan Textile Factory The Wind Road South Middle, Gaotang County Economic Development Zone, Liaocheng City, ShanDong Province, China	Deltamethrin
19	Panda Net 2.0 LLIN	LIFE IDEAS Biological Technology Co., Ltd, China	Life Ideas Biological Technology Co., Ltd, (Building 1#) No.6-4, North Jianda Road, Jiangmen 529000,China	Life Ideas Biological Technology Co., Ltd., Chengxi Industrial District, Hutang Town, Changzhou City, Jiangsu,China	Deltamethrin
20	Tsara Boost	NRS Moon netting FZE, Dubai	Sheikh Noor-ud-Din & Sons 4 km, Kanha Kacha Road, off Ferozepur Road, Lahore, Pakistan	Sunpack Hanjiang Road 368#, Changzhou, Jiangsu, China	Deltamethrin, Piperonyl butoxide
ITN treatment Kits					
1	Fendona 10 SC	BASF SE, Germany	Tagros, India; Bayer Vapi, India		Alpha-cypermethrin
2	Fendona 6 SC	BASF SE, Germany	Tagros, India; Bayer Vapi, India		Alpha-cypermethrin
3	Pendulum 6 SC	Gharda Chemicals Limited, India	D-1/2, B-1/7, F-1/1, MIDC, Lote Parshuram, Talika-Khed, Distt-Ratnagiri Maharashtra 415722, India		Alpha-cypermethrin
4	Pendulum 10 SC	Gharda Chemicals Limited, India	D-1/2, B-1/7, F-1/1, MIDC, Lote Parshuram, Talika-Khed, Distt-Ratnagiri Maharashtra 415722, India		Alpha-cypermethrin
5	ICON CS–ITN Kit	Syngenta Crop Protection AG, Switzerland (Parent; ChemChina)	Syngenta Seneffe BV Syngenta Seneffe, Rue de Tyberchamps 37, B-7180, Seneffe, Belgium and Syngenta Hellas S.A. 2nd km Kinotiki odos Enofyta.Ag. Thomas 32011 Enofyta Viotias Greece		Lambda-cyhalothrin
6	Vectron 10EW	Mitsui Chemicals Agro, Inc, Japan	Utsunomiya Chemical Industry Co., Ltd Shinshiro Factory, 11-4 Ihara, Oomi, Shinshiro-shi, Aichi 441-1315, Japan		Etofenprox

**Fig. 4:**
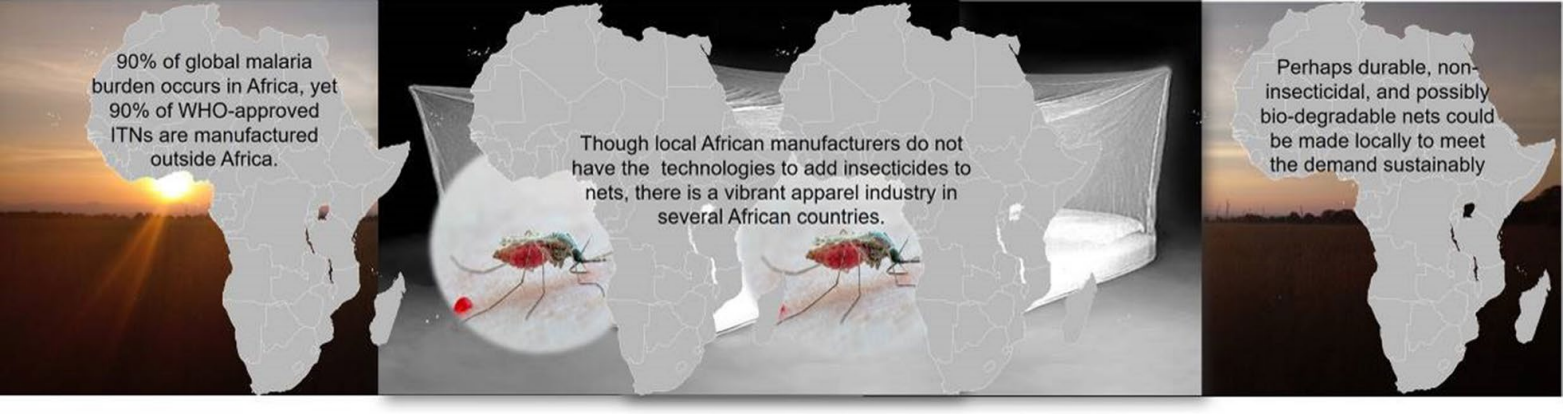
Though ~ 90% of malaria burden occurs in Africa, 17 of the 20 WHO-prequalified nets are manufactured outside Africa (at at 2020), as local manufacturers lack technologies to add insecticides to nets, or produce nets at competitive pricing and scale. It is conceivable that durable, non-insecticidal would be readily manufactured locally, as Africa already has strong apparel-manufacturing industries. Moreover, instead of the non-biodegradable fibres such as polyethylene in many current LLINs, the African nets could possibly be made of cotton or other bio-degradable fibres

In 2017, just 11 countries accounted for 70% of the 219 million malaria cases and 435,000 deaths globally [30]. Ten were in sub-Saharan Africa, and the other was India. Nigeria, Democratic Republic of Congo, Mozambique, Uganda, Burkina Faso, Ghana, Niger and Cameroon together constituted 60% of all malaria cases globally [30]. Unfortunately, even these high burden countries do not have sustainable local production of the WHO-approved nets, and instead rely primarily on importation. Prevailing procurement practices by governments and funding partners, such as the Global Fund for AIDS, Tuberculosis & Malaria (GFATM) and US President’s Malaria Initiative require WHO prequalification and constitute bulk tenders requiring large-scale manufacturing and deliveries often beyond reach of local manufacturers. Besides, small-and medium-sized manufacturers often miss the essential technologies for impregnating or coating nets with insecticides. As a result, while many malaria endemic countries already have vibrant textile industries, manufacturing ITNs at competitive quality, pricing and scale remain challenging (Fig. 4).

Thankfully, there have already been a few outstanding exceptions e.g. in Tanzania where Olyset^®^ nets are produced with royalty-free technology from the Japanese manufacturer, Sumitomo Chemicals, which ships polyethylene pellets containing permethrin for end-manufacturing and sewing by A to Z Textiles in, Tanzania [207]. A to Z already had capacity of 30 million LLINs annually by 2010, and remains the largest local LLIN producer in Africa. Another example is the DawaPlus Made in Africa Program, a cut-to-pack operation in Nigeria, where completed LLIN fabric is shipped from Pakistan to a local dealer (Rosie’s Textiles), which cuts and packs finished DawaPlus nets [208]. In 2017, this programme made and distributed 425,000 LLINs in Nigeria. More recently, Rwanda also began producing ITNs locally in a bid to reduce procurement costs and guarantee access; and the locally-produced nets are scheduled for distribution beginning 2020 [192].

While local manufacturing may itself not immediately guarantee quality, it will likely encourage more sustainable innovation platforms, which countries could rely upon in the event that global financing and pool procurement for ITNs dwindle. Given the potential of untreated but durable nets, relaxing the universal requirement of insecticides as a core component in nets, and instead focusing on physical integrity, acceptability, affordability and mass access could be a useful first step, potentially rejuvenating local enterprises. Besides, endemic countries and the international community should encourage policies that incentivize creativity and manufacturing of high-quality fashionable nets, even if non-insecticidal, based on local people’s preferences so as to maximize use. Care must be taken to ensure that local production does not excessively increase costs or compromise quality of these untreated nets, thereby reducing the cost-effectiveness. Similarly, decision-makers should ensure that the local production does not influence decision-making, e.g. by countries preferentially opting for locally-produced nets over more cost-effective products from outside, and that post-approval quality remains high.

In a 2016 review, titled “averting a malaria disaster” [175], Hemingway et al. warned that new insecticides would not arrive in the market until after 5 years. There are also no guarantees that any new insecticides would not be used as monotherapies, which could lead to rapid evolution of resistance. Countries should, therefore, plan beyond the insecticide-dependent war on malaria, and concurrently develop inclusive options, possibly including long-lasting untreated nets. In-country solutions are likely to foster sustainability while also responding to user preferences.

## Pyrethroid-treated bed nets remain critical for tackling transmission rebounds, outbreaks and emergencies

The evidence above acknowledges the major impacts of insecticide net treatments in historical contexts, but questions whether the strategy of adding insecticides onto bed nets is still relevant or cost-effective, and whether it should remain a universal requirement. Beyond this, there are still some situations where insecticidal nets remain highly valuable, and where countries should consider stockpiling the new generation or multi-active LLINs for use. Some of these are listed below (list not conclusive):

First are areas undergoing malaria transmission rebounds mediated by vectors that primarily bite humans indoors and are sufficiently susceptible to the ITN treatments. The historical coincidence of declining populations of *An. gambiae s.s*. and the scale-up of ITNs in some African villages [209] suggests that the nets could offer essential protection against these vectors if these rebounded. *Anopheles funestus* is also known to rebound after withdrawal of insecticide use [210, 211]. Such rebounds, if mediated by susceptible mosquitoes could be effectively controlled if stockpiles of LLINs managed at district or regional level are available. Instead of the current one-size-fits-all approach, it is possible that targeted deliveries of ITNs, in particular those with multiple actives of synergists, to areas with the greatest risk may achieve higher impact that non-targeted roll-out of pyrethroid-only nets.

Second is epidemic situations. If other high value interventions like IRS are not readily available or are logistically challenging to deploy, second generation LLINs (e.g. PBO-Nets) could be highly effective for epidemics even if the synergists do not last very long on the LLINs. Districts prone to epidemics should, therefore, stockpile the LLINs, even as general control programmes rely on high coverage with simply durable nets. Lastly, emergency situations, refugee camps or camps of internally displaced persons affected by natural disasters such as floods will need rapid response, achievable using stockpiled LLINs. However, even in these situations, alternative insecticidal applications, such as IRS with non-pyrethroids may appropriate if combined with long-lasting untreated nets.

## Conclusions

The purpose of this paper was not to discredit ITNs, but to illustrate that singular focus on their insecticidal content can hinder further innovation and sustainability around bed nets and malaria prevention. It is increasingly crucial to provide context-appropriate solutions and to acknowledge that long-lasting untreated nets can be impactful in most contemporary settings, particularly if LLINs are expensive or distributions limited. The article makes a case for the importance of properties other than bio-efficacy, (e.g. physical barrier effects leading to bite prevention, consistent use and high population-level coverage) as being also important. The overriding message is that intact nets, if consistently used, can offer substantial benefits whether or not they kill mosquitoes. Whether the benefits would be equivalent to those provided by insecticidal nets remains an important question to pursue.

Community-level protection historically accruable from the mass-killing effects of ITNs in areas where *Anopheles* populations were susceptible have been largely lost due to resistance but these gaps can be filled by maintaining high net coverage, even if these nets only prevent biting. As a result, overall effectiveness of nets is not always attenuated by pyrethroid resistance. This raises the question as to whether the nets must always be insecticidal. The best way to maximize benefits of the nets is, therefore, to maintain them as intact and durable, and to promote consistent use at high coverage.

Another important concern is local availability of effective bed nets in countries where they are most needed. Though ~ 90% of malaria burden occurs in Africa, most World Health Organization-prequalified nets are manufactured outside Africa, since many local manufacturers lack capacity to produce high-quality ITNs at competitive scale and pricing. By relaxing conditions for insecticides on nets, it is conceivable that non-insecticidal, but durable, and possibly bio-degradable nets, could be readily manufactured locally. A rejuvenated in-country production for durable untreated nets in endemic countries would effectively boost and sustain access.

Lastly, it is important to compare epidemiological outcomes when using untreated nets or treated nets with similar-quality fabric in different resistant settings. Recognizing the potential ethical concerns arising from ITNs being the current best practice, the studies should be done under careful public health supervision and malaria case management. Where feasible, studies may also compare different net fibers and knitting patterns relevant to attributes such as “softness” and costs. This should be followed by mathematical simulations of the potential of untreated nets as well as cost-effectiveness analyses to help countries determine which net product (untreated nets, pyrethroid-treated nets, PBO-based nets, or dual-active nets) is best for each setting.

## Data Availability

Not applicable.
